# Trastuzumab Modulates the Protein Cargo of Extracellular Vesicles Released by ERBB2^+^ Breast Cancer Cells

**DOI:** 10.3390/membranes11030199

**Published:** 2021-03-12

**Authors:** Silvia Marconi, Sara Santamaria, Martina Bartolucci, Sara Stigliani, Cinzia Aiello, Maria Cristina Gagliani, Grazia Bellese, Andrea Petretto, Katia Cortese, Patrizio Castagnola

**Affiliations:** 1 DIMES, Department of Experimental Medicine, Human Anatomy, Università di Genova, 16132 Genova, Italy; silviamarconism@libero.it (S.M.); 3897061@studenti.unige.it (S.S.); gagliani@unige.it (M.C.G.); 53346@unige.it (G.B.); cortesek@unige.it (K.C.); 2 Core Facilities-Proteomics Laboratory, Istituto Giannina Gaslini, 16147 Genova, Italy; smartibartolucci@gmail.com (M.B.); a.petretto@gmail.com (A.P.); 3 IRCCS Ospedale Policlinico San Martino, 16132 Genova, Italy; Sara.stigliani@hsanmartino.it (S.S.); cinzia.aiello@hsanmartino.it (C.A.)

**Keywords:** trastuzumab, HER2, extracellular vesicles, breast cancer, proteomic analysis, immunoelectron microscopy, TOP1, CD63, mitochondria

## Abstract

Cancers overexpressing the ERBB2 oncogene are aggressive and associated with a poor prognosis. Trastuzumab is an ERBB2 specific recombinant antibody employed for the treatment of these diseases since it blocks ERBB2 signaling causing growth arrest and survival inhibition. While the effects of Trastuzumab on ERBB2 cancer cells are well known, those on the extracellular vesicles (EVs) released from these cells are scarce. This study focused on ERBB2^+^ breast cancer cells and aimed to establish what type of EVs they release and whether Trastuzumab affects their morphology and molecular composition. To these aims, we performed immunoelectron microscopy, immunoblot, and high-resolution mass spectrometry analyses on EVs purified by differential centrifugation of culture supernatant. Here, we show that EVs released from ERBB2^+^ breast cancer cells are polymorphic in size and appearance and that ERBB2 is preferentially associated with large (120 nm) EVs. Moreover, we report that Trastuzumab (Tz) induces the expression of a specific glycosylated 50 kDa isoform of the CD63 tetraspanin and modulates the expression of 51 EVs proteins, including TOP1. Because these proteins are functionally associated with organelle organization, cytokinesis, and response to lipids, we suggest that Tz may influence these cellular processes in target cells at distant sites via modified EVs.

## 1. Introduction

ERBB2 is a transmembrane protein that belongs to the ERBB family of growth factor receptors. At variance from other members of the family, ERBB2 has not a known ligand and therefore is classified as an orphan receptor. The ERBB2 structural conformation allows ligand-independent dimerization and signaling activity. ERBB2 overexpression, caused most often by gene amplification, may lead to oncogenic transformation in several human cell types, which in turn generate breast, ovarian, bladder, gastric, and several other tumors. In fact, ERBB2 signaling promotes key cellular processes such as proliferation, survival, migration, invasion, and angiogenesis, and therefore, its overexpression in tumors is also associated with a poor prognosis [1,2]. For all these reasons, targeting ERBB2 and blocking its signaling activity with a specific humanized antibody named Trastuzumab (Tz) is still a milestone in the therapy of ERBB2 overexpressing (ERBB2^+^) tumors [3].

Extracellular vesicles EVs are released by many cells in the extracellular environment. Based on their biogenesis, they can be distinguished into three classes, namely, (i) exosomes with a diameter of 50–150 nm, forming as intraluminal vesicles (ILVs) within late endosomal organelles known as multivesicular bodies (MVBs), (ii) larger EVs called microvesicles (MVs, 100–1000 nm) shedding from the plasma membrane, and (iii) apoptotic vesicles, representing end death products (100–5000 nm). In particular, exosomes are the foremost studied particles for their involvement in EV different biological functions [4]. Tumor cells both in vitro and in vivo release in the environment EVs that have been implicated in intercellular communication with target cells at distant sites mediating a number of processes that promote tumor metastasis and progression, including microenvironment modulation, migration, invasion, angiogenesis, epigenetic modulation, immune evasion and drug resistance [5,6,7]. ERBB2^+^ cancer cells release EVs displaying this orphan receptor on the membrane. This may contribute to the resistance of ERBB2 tumors to Tz. In fact, it has been shown that EV carrying ERBB2 binds Tz and inhibits its antiproliferative activity on ERBB2 breast cancer cells, likely by a sequestering mechanism [8]. A similar mechanism of resistance to the therapeutic monoclonal Rituximab has been reported for EVs released from malignant lymphoma displaying the CD20 protein [9]. While there is a wealth of knowledge concerning the effects of Tz on ERBB2^+^ cancer cells and on EV biogenesis, structure, and functions, less is known about possible modifications induced by Tz on EVs released by ERBB2^+^ cells. To fill this gap of knowledge, we focused on cell line models of ERBB2^+^ breast cancer (BCa) and used a multidisciplinary approach, including transmission electron microscopy (TEM), biochemistry, and high-resolution mass spectrometry. Our results show that EVs released by ERBB2^+^ cell lines fall in three morphological classes, ERBB2 is preferentially present in larger EVs, and that Tz modifies proteins carried by EVs with a possible impact on processes such as organelle organization, cytokinesis, and response to lipids in target cells.

## 2. Materials and Methods

### 2.1. Cell Lines and Cell Culture Reagents

The breast cancer (BCa) cell lines BT474, SKBR-3, and MDA-MD-361 were obtained from Banca Biologica and Cell Factory in IRCCS Ospedale Policlinico San Martino (Genova, Italy) affiliated to the European Culture Collection’s Organization. Culture media for routine cell expansion was Dulbecco’s modified Eagle medium (DMEM) high glucose supplemented with 1% glutamine, penicillin, and streptomycin, and 10% heat-inactivated fetal bovine serum for BT474 and SKBR-3 or 20% for MDA-MD-361 (Euroclone S.p.A, Pero, Italy). EVs production medium was prepared as reported by Thery et al. [10]. Cultures were performed at 37 °C in humidified 5% CO_2_ atmosphere. Normal human immunoglobulins G IgGs (CLS Behring, King of Prussia, PA, USA) and Tz (Genentech-Roche, South San Francisco, CA, USA) were dissolved with saline solution with 0.9% NaCl in a stock concentration of 21 mg/mL, donated by the Unità Farmaci Antiblastici of the IRCCS Ospedale Policlinico San Martino. Tz was used at a concentration of 10 µg/mL. Control cells were cultured with normal human IgGs at the same concentrations used for Tz.

### 2.2. EV Purification

After 72 h of treatment, EV purification from a cell-conditioned medium was performed as described previously [8]. Briefly, the day before treatment cells were detached with trypsin in phosphate-buffered saline (PBS), counted, and seeded at 5.3 × 10^4^ cells/cm^2^. At time 0, cells were washed three times with PBS before the addition of EVs production medium containing human IgGs (control condition) or Tz, both at 10 µg/mL. After 72 h the conditioned medium was subjected to two sequential centrifugations at 300 *g* for 10 min and at 2000 *g* for 30 min followed by filtration through 0.22 µm filters to eliminate cell debris. Filtered supernatants were centrifuged in polyallomer tubes at 100,000 *g* for two hours at 4 °C using an SW28 or SW41 rotor and the Optima XPN-100 Ultracentrifuge (Beckman Coulter Inc., Fullerton, CA, USA). The EV-containing pellets were dissolved in PBS and concentrated at 100,000 *g* for 60 min using a TLA 100.3 rotor and the TL100 Ultracentrifuge (Beckman Coulter Inc., Brea, CA, USA). Pelleted EVs were dissolved in PBS. 

### 2.3. Immunoblot and Biochemical Assays

Protein concentration in cell lysates and EVs preparations was established by using a colorimetric detection and quantitation assay based on bicinchoninic acid (BCA) (Thermofisher Scientific, Waltham, MA, USA). When indicated cell lysates were digested with N-Glycosidase F (Roche Diagnostics, Monza, Italy) according to the manufacturer’s instruction. Proteins were subjected to electrophoresis on Bolt 4–12% Bis Tris Plus gels (Thermofisher Scientific) and blotted on a polyvinylidene fluoride PVDF membrane. Primary antibodies used in this study are listed in Table S1 (Supplementary Materials). Detection was performed using horseradish peroxidase conjugated anti-mouse or anti-rabbit antibodies (Thermofisher Scientific, Waltham, MA, USA) and with enhanced chemiluminescent (ECL) Detection Reagent from BioRad (Hercules, CA, USA), according to manufacturer’s protocol. ECL signals were imaged by the Nine Alliance, Uvitec (Cambridge, United Kingdom), gel documentation apparatus. 

### 2.4. Mitochondrial Morphological Analysis by MitoTracker Red Labeling

Cells were cultured in control conditions or in presence of Tz for 72 h and labeled with MitoTracker Red (Thermofisher Scientific, Waltham, MA, USA) according to the manufacturer’ instructions. Cells were fixed and fluorescent images were acquired using Axio Imager A2M microscope (Carl Zeiss, Jena, Germany). Mitochondrial size and area were measured by using the object analyzer advanced tool of Huygens Professional version X11 (http://svi.nl, accessed on 11 March 2021) (Scientific Volume Imaging, Hilversum, The Netherlands).

### 2.5. TEM Imaging of Cells and EV Immunolabeling

Cells were incubated with 5 nm gold-conjugated bovine serum albumin (BSA) at optical density (OD) 5 (purchased from Cell Microscopy Core, Department of Cell Biology, University Medical Center Utrecht, Utrecht, The Netherlands) for 2 h at 37 °C and then washed twice in 0.1 M cacodylate buffer and fixed in 0.1 M cacodylate buffer, 2.5% glutaraldehyde (Electron Microscopy Science, Hatfield, PA, USA), for 1 h at room temperature. Cells were postfixed in 1% osmium tetroxide for 2 h and 1% aqueous uranyl acetate for 1 h. Samples were then dehydrated through a graded ethanol series and flat embedded in resin (Poly-Bed; Polysciences, Inc., Warrington, PA, USA) for 24 h at 60 °C. Ultrathin sections (50 nm) were cut parallel to the substrate and counterstained with 5% uranyl acetate in 50% ethanol.

EVs preparations resuspended in PBS were fixed with an equal volume of 2% paraformaldehyde in 0.1 M phosphate buffer (pH 7.4) and then adsorbed for 10 min to formvar-carbon coated copper grids by floating the grids on 5 µL drops on parafilm. Grids with bound vesicles were then rinsed in PBS and negatively stained with 2% uranyl acetate for 5 min at room temperature. Grids were embedded in 2.5% methylcellulose to improve preservation and air-dried before imaging. For immunolabeling of EVs, 5 µL of a concentrated solution of EVs were dropped on Formvar-coated copper grids and incubated for 20 min at room temperature. After 5 min wash in PBS, the EVs were fixed in 1% Glutaraldehyde for 5 min. A second wash in PBS and two passages in 0.2% Glycine in PBS for 5 min each were performed to saturate non-specific reactive sites. After a passage in 1% BSA in PBS for 5 min, the grids were incubated with anti-ERBB2 (9G6, sc-08 Santa Cruz Biotechnology, Dallas, TX, USA) in 1% BSA in PBS for 30 min at room temperature. Unbound antibody was removed by 4 × washes in 1% BSA of 2 min each. ERBB2 was then revealed by incubation with Protein A conjugated with 10 nm colloidal gold (PAG, purchased from Cell Microscopy Core Department of Cell Biology University Medical Center Utrecht, Utrecht, The Netherlands). Excess PAG was eliminated by four washes in 1% BSA of 2 min each, which were followed by a further passage in 1% Glutaraldehyde for 5 min, a wash in PBS for 5 min, and two washes in distilled water. Samples were counterstained with 2% uranyl acetate in 0.15 M oxalic acid for 5 min at room temperature. A final incubation in Methylcellulose 1.8% in uranyl acetate 4% for 5 min at room temperature was performed to enhance contrast and allow the formation of a protective layer on the samples. Electron micrographs were obtained with a Hitachi 7800 120 Kv electron microscope (Hitachi, Tokyo, Japan) using a Megaview 3 digital camera and Radius software (EMSIS, Muenster, Germany). Morphometry analysis of the size of EVs was measured on 10 randomly taken micrographs at 40,000× magnification for each condition and was calculated using the arbitrary line function embedded in the measurement dialog box of Radius software. To visualize EVs size distribution, the results were plotted as a boxplot.

### 2.6. Sample Preparation and Mass Spectrometer Setup

Samples were lysed, reduced, and alkylated in 120 µL 6M Guanidine, 10 mM TCEP, 4 mM CAA, 100 mM Tris pH 8. Then proteins were extracted with the principal component analysis method [11]. Briefly, 4 µL of carboxyl-coated magnetic beads were added to each sample, and proteins were induced to aggregate on the beads with the addition of 70% ACN. The beads washed with 1 mL ACN and 1 mL 70% Ethanol were digested O.N. at 37 °C with 0.7 µg trypsin and 0.3 µg LysC. Digested samples were loaded onto StageTips [12]. The resulting peptides were analyzed by a nano-UHPLC-MS/MS system using an Ultimate 3000 RSLC coupled to an Orbitrap Velos Pro mass spectrometer (Thermo Scientific Instrument, Waltham, MA, USA) in positive ionization mode. Elution was performed with an EASY spray column (75 μm × 50 cm, 2 μm particle size, Thermo Scientific) at a flow rate of 250 nL/min with a 200 min non-linear gradient of 5–45% solution B (80% acetonitrile, 20% H_2_O, 5% dimethylsulfoxid and 0.1% formic acid). MS scans were performed in the Orbitrap at a resolution of 60,000 between 375 and 1500 *m*/*z* using a maximal ion injection time of 50 ms. The automatic gain control was set to 1,000,000 ions. The analysis was conducted in data-dependent acquisition mode with alternating MS and MS/MS experiments. A maximum of 10 MS/MS experiments were triggered per MS scan. MS/MS spectra were acquired in the linear ion trap (rapid scan mode) after collision-induced dissociation (CID) fragmentation at a collision energy of 35% and an AGC target of 10,000. Dynamic Exclusion was set at 30 s. Data processing was performed by MaxQuant [13] software version 1.6.10.43. A false discovery rate was set at 0.01 for the identification of proteins and a minimum of 6 amino acids was required for peptide identification. The Andromeda engine was used to search MS/MS spectra against the Uniprot human database (release UP000005640_9606 April 2019). Algorithm MaxLFQ was chosen for the protein quantification with the activated option “match between runs” to reduce the number of the missing proteins. The intensity values were extracted and statistically evaluated using the ProteinGroup Table and Perseus software version 1.6.8.0 [14]. The mass spectrometry proteomics data have been deposited to the ProteomeXchange Consortium via the PRIDE [15] partner repository with the dataset identifier PXD024276. 

## 3. Results

### 3.1. SKBR-3 Cells Release Three Morphological Classes of EVs

**Figure 1:**
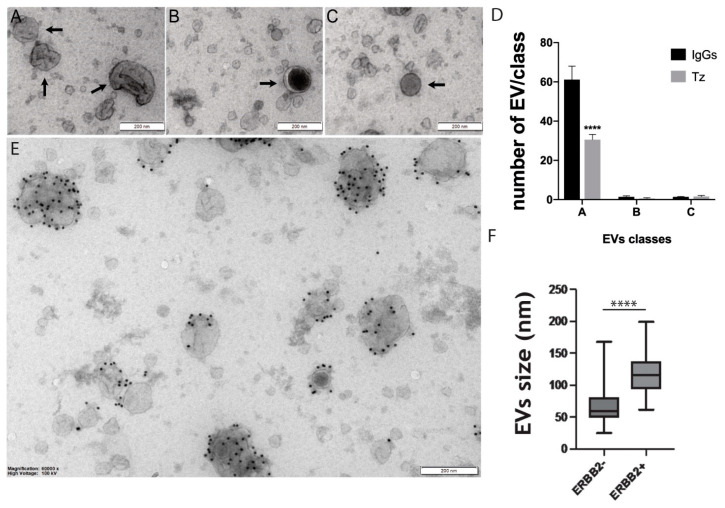
Representative transmission electron microscopy (TEM) images of extracellular vesicles (EVs) purified from conditioned media of SKBR-3 cells. Three classes of EVs, which differ by size and ultrastructure can be identified: (**A**) included 30–300 nm cup-shaped EVs indicated by arrows; (**B**) the 30–200 nm round EVs with a dark electron-dense core and a clear peripheral region indicated by an arrow; (**C**) the 30–80 nm round and homogeneously light electro-dense EVs indicated by an arrow. (**D**) Grouped histogram showing the number of EVs falling into each class in trastuzumab (Tz)-treated compared to immunoglobulins G (IgGs) treated SKBR3 cells and counted on 10 random micrographs at 30,000× magnification. Bars represent the mean ± SEM. **** *p* < 0.0001 (2-way ANOVA test). (**E**) ERBB2 specific immunogold staining of purified EVs. ERBB2 was labeled with anti-ERBB2 antibody (9G6, sc-08, Santa Cruz) and protein A gold conjugated (10 nm colloidal gold) was used to reveal the primary antibody. (**F**) Box-and-whiskers boxplot showing size distribution ERBB2^+^ and ERBB2^−^ EVs purified from SKBR-3 cells, **** *p* < 0.0001 (Student’s *t*-test). Magnification bars = 200 nm.

To characterize EVs released in the culture media by the ERBB2^+^ BCa cell line SKBR-3, we performed an ultrastructural analysis by TEM. The first and most abundant class (A) included cup-shaped EVs with sizes ranging from 30–300 nm. The second one (B) with a 30–200 nm in diameter, showed a round shape with a dark electron-dense core surrounded by a clear peripheral region. Lastly, the third class (C) included a more homogeneously lighter electron-dense and a round-shaped subpopulation of EVs characterized by a diameter of 30–80 nm (Figure 1A–D). We then performed a morphometry analysis to assess whether Tz modulates the release of the overall population and/or a specific subset of EVs. Results showed that Tz significantly decreased the number of class (A) cup-shaped subsets of EVs (Figure 1D). Next, we assessed the association of ERBB2 with a particular subset of these EVs. We performed an immuno-electron microscopy analysis using an antibody that recognizes the N-terminal domain of this receptor (9G6). Results showed that ERBB2 is preferentially expressed by larger class (A) EVs with a median diameter of about 120 nm (*p* < 0.0001) (Figure 1E,F). The association of ERBB2 to large EVs is maintained after Tz treatment (not shown).

### 3.2. Tz Treated ERBB2^+^ BCa Cells Release EVs Expressing the 50 kDa Isoform of the CD63 Tetraspanin

**Figure 2:**
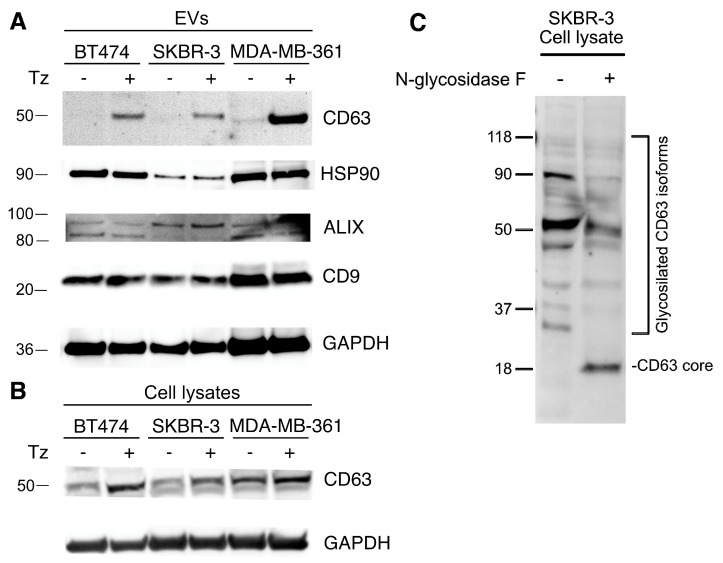
Immunoblot analyses of ERBB2^+^ cancer cell lines, lysates, and EVs purified from conditioned media. Cells were either treated with human normal IgGs (Tz− condition) or with 10 µg/mL of Tz (Tz+ condition) for 72 h. Migration of molecular mass standards expressed in kDa, are indicated on the left. (**A**) Immunoblot analysis of EVs purified from conditioned media of BT474, SKBR-3, and MDA-MB-361 cell lines. Antibody tests are indicated on the right. GAPDH was used as the loading control. (**B**) Immunoblot analysis of the expression levels of the CD63 50 kDa isoform in BT474, SKBR-3, and MDA-MB-361 cell layers. GAPDH was used as the loading control. (**C**) Immunoblot analysis of cell lysates treated with reaction buffer (N-glycosidase F− condition) or with buffer plus enzyme (N-glycosidase F+ condition). Lysates were obtained from SKBR-3 cells treated with Tz. Signals from glycosylated CD36 isoforms and the 18 kDa CD63 core protein are indicated on the right.

To better characterize the EVs released from SKBR-3 cells we performed immunoblot analysis with antibodies recognizing the HSP90, which stabilize the ERBB2 receptor, and several EVs markers such as PDCD6IP/Alix, CD9, and CD63. GAPDH, which is a constitutively expressed protein in EVs, was used as the loading control. The immunoblot analysis showed that the EVs express the HSP90 chaperone along with CD9 and Alix and that Tz treatment did not induce major changes in the levels of these proteins. In contrast, a faint signal corresponding to a 50 kDa CD63 isoform was observed only in EVs purified from Tz treated SKBR-3. A similar Tz-dependent expression was displayed by EVs from BT474 and MDA-MB-361 cells (Figure 2A). A Tz-dependent expression of the CD63 50 kDa isoform reflected the results obtained in the cell lysates obtained from the three ERBB2+ BCa cell lines (Figure 2B). We hypothesize that this 50 kDa isoform is the result of specific glycosylation. To test this hypothesis, we performed an N-glycosidase F treatment of lysates of SKBR-3 treated with Tz followed by immunoblot analysis with the CD63 antibody. Results showed that N-glycosidase F caused a reduction of the intensity of the 50 kDa band and the appearance of an 18 kDa band, corresponding to the CD63 core protein, confirming our hypothesis (Figure 2C).

### 3.3. Tz Modulates the Expression of Proteins Associated with EVs of SKBR-3 Cells

**Figure 3:**
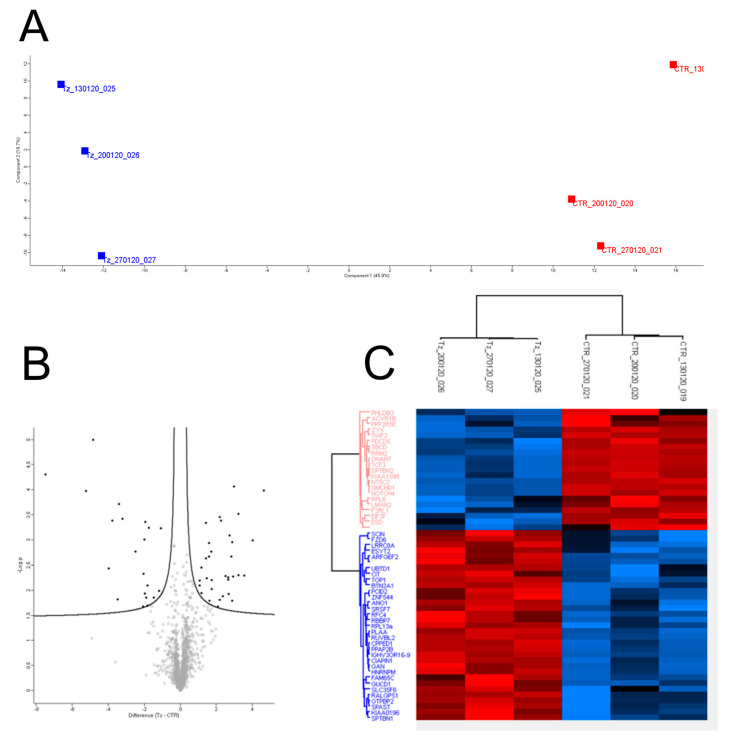
Principal component analysis, volcano plot and unsupervised hierarchical clustering analysis of proteins associated with EVs released from Tz treated or control SKBR-3 cells. (**A**) Two-dimensional scatter plot of the principal component analysis of EVs released from Tz treated (blue dots) or control IgGs treated SKBR-3 cells (red dots). (**B**) Volcano plot representation of differentially expressed proteins. (**C**) Unsupervised hierarchical-clustered heatmap of 52 proteins identified by Multiple-samples test ANOVA performed on the EVs purified from the SKBR-3 cell line. The amount of each protein in individual samples is represented by the color scheme in which red and blue indicate high and low expression of proteins, respectively. Three independent biological replicates of cells treated with Tz or IgGs (control, CTR) are shown. Proteins are clustered into two groups according to their expression value. In particular, 32 were up-regulated in EVs from Tz treated cells, while 21 were upregulated in EVs from control cells.

To gain insight on the protein associated with EVs produced by SKBR-3 cells under Tz treatment, we used a proteomic approach based on high-resolution mass spectrometry on EVs purified from the SKBR-3 cell line treated with Tz or with IgGs for 72 h, in triplicate experiments. A range of 1478–1483 proteins was identified in the three-control EV samples, while 1503–1520 proteins were identified in the three Tz-treated EV samples. To reveal differences or similarities between the two treatments, we performed principal component analysis (PCA) and hierarchical clustering analysis. The PCA analysis showed that the EV-associated proteins are separated according to treatments in two groups (Figure 3A) and the volcano plot in Figure 3B shows differentially expressed proteins. In particular, 32 proteins (including the IgGs heavy chain of the Tz added in the treatment) were upregulated while 20 proteins were downregulated in EVs derived from Tz treated SKBR-3 compared to controls (t-test S0 = 0.1 and false discovery rate FDR = 0.05). A list of these 51 proteins (which obviously excluded the IgGs heavy chain) regulated by Tz in EVs from SKBR-3 is provided (Table S2). Unsupervised hierarchical clustering analysis showed that the modulated proteins are clustered in two well-defined groups, which is in agreement with the PCA analysis (Figure 3C). To gain insights into possible functions associated with the two protein groups, we performed a bioinformatic analysis using the HumanBase functional module discovery tool available at https://hb.flatironinstitute.org/ (accessed on 11 March 2021), which perform data-driven predictions of gene functions [16,17,18,19]. 

**Figure 4:**
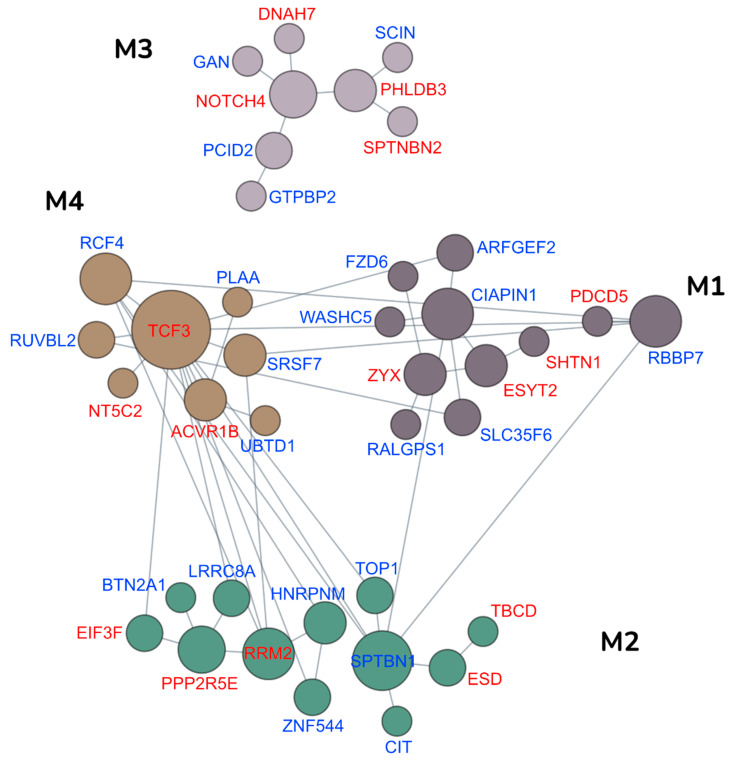
Functional networks of Tz modulated proteins in EVs released from SKBR-3 cells. The biological processes associated with the Tz modulated protein signature associated with EVs released from SKBR-3 cells proteins were characterized by projecting these signature proteins onto mammary epithelium-specific functional networks at HumanBase (https://hb.flatironinstitute.org, accessed on 11 March 2021). These network modules represent genes and their interactions in biological processes and pathways active in mammary epithelium. The most representative biological processes enriched within each module are as follows: M1 mitochondrial membrane organization, M2 mitotic cytokinesis, M3 negative regulation of organelle organization, and M4 cellular response to lipid. The protein expression is represented by the color scheme in which blue and red protein symbols indicate high and low expression of the corresponding proteins in EVs from Tz treated cells, respectively. The diameter of a node corresponds to the number of connections to other nodes.

In particular, this analysis was focused on data obtained from the mammary epithelium and showed that the genes encoding for the 51 proteins regulated by Tz in EVs belong to four distinct functional modules—M1 mitochondrial membrane organization, M2 mitotic cytokinesis, M3 negative regulation of organelle organization, and M4 cellular response to lipid (Q value < 0.01) (Figure 4). 

### 3.4. Tz Triggers Mitochondrial Alterations in SKBR-3 Cells

**Figure 5:**
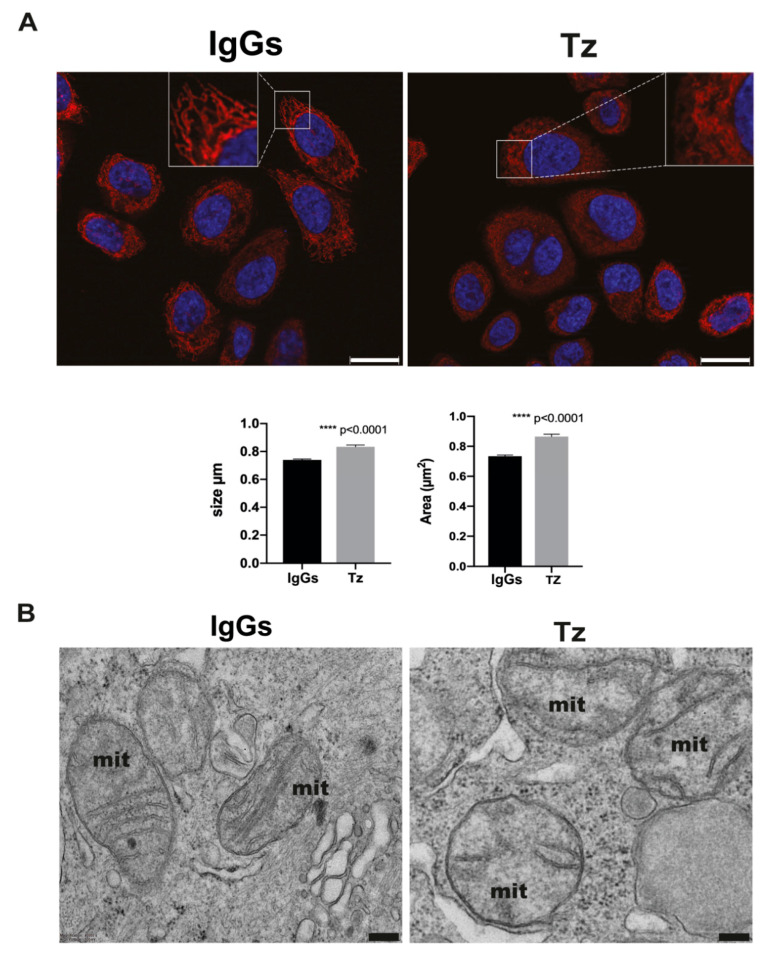
Fluorescence and TEM imaging of SKBR-3 cell mitochondria. (**A**) Mitochondria stained with MitoTracker Red display altered morphology (magnification insets) and increased size and area in cells treated with Tz compared to controls (IgGs). **** *p* < 0.0001. Size bar = 20 µm. (**B**) TEM micrographs showing mitochondria in Tz-treated cells with abnormal architecture and reduced number of cristae compared to controls (IgGs). Size bars: 200 nm.

Because the most represented functional module found through our bioinformatic analysis of the EV-associated proteins modulated by Tz was related to mitochondrial membrane organization, we hypothesize that Tz could affect the mitochondrial structure in cancer target cells. In particular, we analyzed the size and area of SKBR-3 mitochondria labeled with the specific fluorescent marker MitoTracker Red and found a statistically significant increase of both of these parameters upon Tz treatment (Figure 5A). These data, indicative of mitochondrial alterations caused by Tz, prompted us to better characterize the mitochondrial phenotype in ERBB2^+^ cells by performing TEM analysis of SKBR-3. Our results showed a reduction of mitochondrial cristae in Tz treated cells compared to controls (Figure 5B).

## 4. Discussion

A great research effort has been recently focused on EVs released from human malignancies because they may potentially contribute to early diagnosis of primary tumors and in the detection of relapse after therapy. However, more information is needed on the effect of anticancer therapy on the structure and composition of EVs released from treated tumors. This study focused on the effects of the drug Tz on EVs released from ERBB2^+^ (BCa) cells using widely used cell lines derived from this tumor type and found that Tz indeed modulates the composition of proteins carried by EVs. Our TEM and immuno-electron microscopy results showed that EVs released from these cells fall in three morphological classes and also determined that ERBB2 is preferentially associated with larger cup-shaped EVs (class A). The presence of these vesicles along with the detection of the exosomal markers CD9, Alix, and CD63 by immunoblot analysis strongly suggests the presence of an exosome subset in these EVs. We found that Tz significantly decreases the release of EVs but has no major impact on all exosomal markers with the notable exception of the 50 kDa CD63 isoform inclusion in the EVs, which was undetectable in those from control cells. Our biochemical data demonstrate that this is a specific glycosylated isoform, which is present also in the cell lysates but whose level of expression in the cells and especially its association to EVs is dependent upon Tz administration to the cells. To our knowledge, this is the first report of a Tz specific effect on posttranslational modification and trafficking of a protein, which in this case is an exosomal marker. Interestingly, CD63 is a member of the tetraspanins superfamily and a key regulator of beta1 integrin signaling and it is involved in the regulation of membrane protein trafficking, reorganization of the actin cytoskeleton, cell adhesion, spreading, and migration [20,21]. Because all these processes are critical for cancer progression, our observation warrants further research on the molecular mechanisms that mediate this Tz effect and possible functional implications of the inclusion of CD63 50 kDa isoform in exosomes released by ERBB2^+^ BCa cells. The fact that CD63 was found in mass spectrometry, but is not significantly modulated, may be due to the highly glycosylated structure of the protein. In fact, glycopeptides have a lower ionization efficiency, due to an increase in negative charge and acidity, which causes an ion suppression effect compared to the higher number of unmodified peptides displaying a more intense ion current. This leads to a measurement less accurate, which may justify the discrepancy with the immunoblot results. However, the analysis of our proteomic data revealed that the influence of the Tz treatment of ERBB2^+^ cells on EVs composition is more extensive. In fact, we found that Tz modulates the abundance in these EVs of 51 proteins, which were associated by a network analysis to four biological processes—mitochondrial membrane organization, organelle organization, cytokinesis, and response to lipid. It is interesting to notice that several studies reported that Tz treatment induces mitochondrial morphological alterations up to disruption of both external and internal membranes leading to mitochondrial dysfunction [22,23,24]. However, these studies were focused on myocardiocytes. Here we show that mitochondria of Tz treated ERBB2^+^ BCa cells are enlarged and display a reduction of the number of cristae. Therefore, we hypothesize that these cells may sort in EVs some components of damaged or dysfunctional mitochondria, perhaps along with components of other damaged organelles, in an effort to dispose of them. Possible effects of these proteins in cells that may eventually collect these EVs in vivo, remain to be investigated. Perhaps more complex and more relevant for a possible role of EVs released from ERBB2^+^ BCa Tz-treated cells on distant target cells are those proteins whose incorporation in EVs is modulated by Tz and that are related to response to lipid and cytokinesis. Among the latter, TOP1, which was also found in EVs from murine BRCA1-deficient tumors [25], may be of particular interest to be evaluated in future studies as an EV-associated biomarker in monitoring breast cancer response to therapy for the following reasons: it functions as an oncogene [26], it is frequently amplified in breast cancer [27], and lastly, because it is a target for deruxtecan, which is effectively delivered to ERBB2^+^ breast cancer cells by Tz as antibody drug conjugate (DS-8201a) [28].

## 5. Conclusions

**Figure 6:**
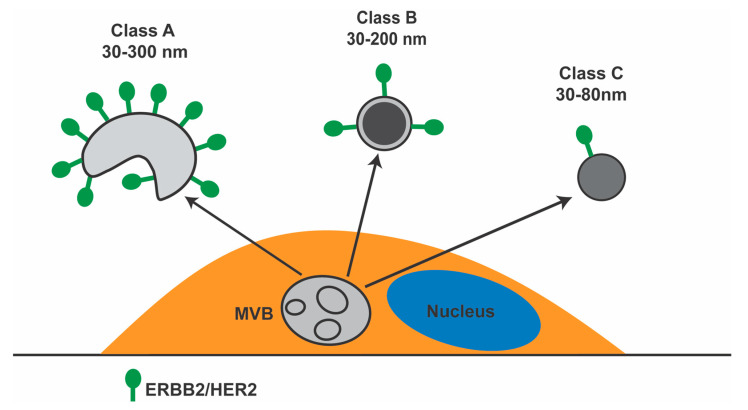
Schematic diagram showing the three main morphological classes of EVs released by SKBR-3 BCa cells. MVB, multivesicular body. ERBB2 on the cell plasma membrane was omitted.

In conclusion, we found that SKBR-3 cells released three main morphological classes of EVs, in which ERBB2 predominantly localizes in class A vesicles (depicted in Figure 6) and that Tz causes enrichment of a 50 kDa CD63 isoform in the EVs purified from all the three ERBB2^+^ cell lines examined—BT474, MDA-MB-361, and SKBR-3. Furthermore, our proteomic study shows that Tz has also an impact on the overall protein cargo of EVs released in the extracellular environment by ERBB2^+^ BCa cells. This cargo reflects at least in part alterations induced by Tz on cell organelle organization and may influence several cellular processes linked to cancer progression in target cells at distant sites.

## Data Availability

The mass spectrometry proteomics data have been deposited to the ProteomeXchange Consortium (https://www.ebi.ac.uk/pride/, accessed on 11 March 2021) with the dataset identifier PXD024276.

## Supplementary Materials

The following are available online at https://www.mdpi.com/2077-0375/11/3/199/s1, Table S1: Antibodies used in this study, Table S2: Proteins regulated by trastuzumab (Tz) in extracellular vesicles (EVs) from SKBR3 cell.
