# Supplementary material for: Trastuzumab Modulates the Protein Cargo of Extracellular Vesicles Released by ERBB2+ Breast Cancer Cells
Source: Membranes (Basel). 2021 Mar 12;11(3):199. doi: 10.3390/membranes11030199 (PMC8000509; doi:10.3390/membranes11030199)
Supplement: Supplementary file 1 [file membranes-11-00199-s001.pdf]

membranes

Supplementary Material: Trastuzumab Modulates the Protein Cargo of Extracellular Vesicles Released by ERBB2+ Breast Cancer Cells

Silvia Marconi, Sara Santamaria, Martina Bartolucci, Sara Stigliani, Cinzia Aiello, Maria Cristina Gagliani, Grazia Bellese, Andrea Petretto, Katia Cortese and Patrizio Castagnola

Table S1. Antibodies used in the study.

Antibody	Catalog number	Manufacturer
Anti-ALIX	sc-271975	Santa Cruz[1]
Anti-CD9	PA5-85955	Thermofisher Scientific[2]
Anti-CD63	sc-15363	Santa Cruz
Anti-ErbB2 (9G6)	sc-08	Santa Cruz
Anti-GAPDH	14C10	Cell signaling[3]
Anti-HSP90	sc-13119	Santa Cruz

[1] Dallas, TX, USA; [2] Waltham, MA, USA; [3] Danvers, MA, USA.

Table S2. Differentially regulated proteins by trastuzumab Tz treatment in extracellular vesicles EVs purified from SKBR-3 cells with a statistically significant p-value resulted from Student's T-test. The gene symbols coding for proteins downregulated by Tz (and hence upregulated in IgG treated cells) are highlighted in red while proteins upregulated by Tz are highlighted in blue.

Official Gene Symbol [1]	Gene product (Protein name)
ACVR1B	Activin A receptor type 1B
ANO1	Anoctamin 1
ARFGEF2	ADP Ribosylation Factor Guanine Nucleotide Exchange Factor 2
BTN2A1	Butyrophilin subfamily 2 member A1
CIAPIN1	Cytokine Induced Apoptosis Inhibitor 1
CIT	Citron Rho-Interacting Serine/Threonine Kinase
CPPED1	Calcineurin Like Phosphoesterase Domain Containing 1
DNAH7	Dynein Axonemal Heavy Chain 7
EIF3F	Eukaryotic translation initiation factor 3 subunit F
ESD	Esterase D
ESYT2	Extended Synaptotagmin 2
F2RL1	F2R Like Trypsin Receptor 1
RIPOR3	RIPOR Family Member 3
FZD6	Frizzled-6
GAN	Gigaxonin
GTPBP2	GTP-binding protein 2
GUCD1	Guanylyl Cyclase Domain Containing 1
HNRNPM	Heterogeneous nuclear ribonucleoprotein M
LMAN2	Lectin, Mannose Binding 2
LRRC8A	Volume-regulated anion channel subunit LRRC8A
NOTCH4	Notch Receptor 4
NT5C2	5'-Nucleotidase, Cytosolic II
PCID2	PCI domain-containing 2
PDCD5	Programmed cell death 5
PHLDB3	Pleckstrin homology like domain family B member 3
PLAA	Phospholipase A2 activating protein

PLPP3	Phospholipid Phosphatase 3
PPP2R5E	Protein Phosphatase 2 Regulatory Subunit B'Epsilon
RALGPS1	Ral GEF With PH Domain And SH3 Binding Motif 1
RBBP7	RB Binding Protein 7, Chromatin Remodeling Factor
RFC4	Replication factor C subunit 4
RPL13A	Ribosomal protein L13a
RPL8	Ribosomal protein L8
RRM2	Ribonucleotide Reductase Regulatory Subunit M2
RUVBL2	RuvB Like AAA ATPase 2
SCIN	Scinderin
SHTN1	Shootin 1
SLC35F6	Solute carrier family 35 member F6
SMCHD1	Structural maintenance of chromosomes flexible hinge domain containing protein 1
SPAST	Spastin
SPTBN1	Spectrin beta, Non-erythrocytic 1
SPTBN2	Spectrin beta, Non-erythrocytic 2
SRSF7	Serine And Arginine Rich Splicing Factor 7
TBCD	Tubulin Folding Cofactor D
TCF3	Transcription factor 3
TOP1	DNA Topoisomerase 1
TWF2	Twinfilin Actin Binding Protein 2
UBTD1	Ubiquitin domain-containing 1
WASHC5	WASH Complex Subunit 5
ZNF544	Zinc finger protein 544
ZYX	Zyxin

[1] https://www.genenames.org (accessed on 11 March 2021).
